# Drugs of Abuse Differentially Alter the Neuronal Excitability of Prefrontal Layer V Pyramidal Cell Subtypes

**DOI:** 10.3389/fncel.2021.703655

**Published:** 2021-08-05

**Authors:** Jonna M. Leyrer-Jackson, Lauren E. Hood, M. Foster Olive

**Affiliations:** Department of Psychology, Arizona State University, Tempe, AZ, United States

**Keywords:** prefrontal cortex, pyramidal cells, hypofrontality, electrophysiology, substance abuse

## Abstract

The medial prefrontal cortex (mPFC) plays an important role in regulating executive functions including reward seeking, task flexibility, and compulsivity. Studies in humans have demonstrated that drugs of abuse, including heroin, cocaine, methamphetamine, and alcohol, alter prefrontal function resulting in the consequential loss of inhibitory control and increased compulsive behaviors, including drug seeking. Within the mPFC, layer V pyramidal cells, which are delineated into two major subtypes (type I and type II, which project to subcortical or commissurally to other cortical regions, respectively), serve as the major output cells which integrate information from other cortical and subcortical regions and mediate executive control. Preclinical studies examining changes in cellular physiology in the mPFC in response to drugs of abuse, especially in regard to layer V pyramidal subtypes, are relatively sparse. In the present study, we aimed to explore how heroin, cocaine, methamphetamine, ethanol, and 3,4-methylenedioxypyrovalerone (MDPV) alter the baseline cellular physiology and excitability properties of layer V pyramidal cell subtypes. Specifically, animals were exposed to experimenter delivered [intraperitoneal (i.p.)] heroin, cocaine, the cocaine-like synthetic cathinone MDPV, methamphetamine, ethanol, or saline as a control once daily for five consecutive days. On the fifth day, whole-cell physiology recordings were conducted from type I and type II layer V pyramidal cells in the mPFC. Changes in cellular excitability, including rheobase (i.e., the amount of injected current required to elicit action potentials), changes in input/output curves, as well as spiking characteristics induced by each substance, were assessed. We found that heroin, cocaine, methamphetamine, and MDPV decreased the excitability of type II cells, whereas ethanol increased the excitability of type I pyramidal cells. Together, these results suggest that heroin, cocaine, MDPV, and methamphetamine reduce mPFC commissural output by reducing type II excitability, while ethanol increases the excitability of type I cells targeting subcortical structures. Thus, separate classes of abused drugs differentially affect layer V pyramidal subtypes in the mPFC, which may ultimately give rise to compulsivity and inappropriate synaptic plasticity underlying substance use disorders.

## Introduction

In humans, the prefrontal cortex (PFC) plays a role in regulating executive functions such as task flexibility, goal-directed behaviors, working memory, and problem-solving, and homologous regions are found within the medial prefrontal cortex (mPFC) of mice. Deficits in prefrontal function often result in loss of inhibitory control, leading to compulsivity and drug-seeking despite serious negative consequences in substance abuse disorders (SUDs). Currently, 20 million Americans suffer from SUDs, including the use of alcohol and other illicit drugs. Compulsive phenotypes have been characterized in both humans (Fattore et al., 2008; Fattore and Melis, 2016) and rodent models of addiction, which coincide with learning, decision-making, and memory impairments (Chen et al., 2013; Giuliano et al., 2018). Along with these behavioral deficits, most drugs of abuse also induce alterations in synaptic physiology, morphology, and regional connectivity within various regions of the brain (Nestler and Lüscher, 2019).

Layer V pyramidal neurons are known to be the major output cells of the mPFC and can be delineated into two major subclasses distinguished by their axonal projection patterns, morphology, receptor expression, and intrinsic electrical properties (Dembrow et al., 2010; Gee et al., 2012; Leyrer-Jackson and Thomas, 2017). These subtypes are defined as type I (pyramidal tract) and type II (intratelencephalic or intracortical) neurons which project to the pontine nuclei of the brainstem or to the contralateral hemisphere of the mPFC, respectively. Type I and type II pyramidal cells can differentially integrate information from subcortical regions (Sherman and Guillery, 1998; Morishima et al., 2011; Dembrow et al., 2015), and modifications to these connections induced by substances of abuse could lead to vast changes in prefrontal integration of information and abnormalities in behaviors mediated by this region. Due to the differences in input connectivity, axonal outputs, and intrinsic excitability properties of PFC output cells, it is likely that drug-induced changes in PFC excitability are highly complex, cell type-specific, and perhaps drug-type specific.

Hypofrontality refers to reductions in prefrontal function that can lead to compulsivity, a prominent feature of substance abuse and many psychiatric disorders (Starcevic and Khazaal, 2017). PFC hypofunction has been observed using functional magnetic resonance imaging in drug-dependent individuals abusing cocaine (Jentsch and Taylor, 1999; Tomasi et al., 2007; Naqvi and Bechara, 2010; Goldstein and Volkow, 2011; Meyer et al., 2014), heroin (Daglish et al., 2001; Rapeli et al., 2006; Fu et al., 2008; Yuan et al., 2009), and methamphetamine (Paulus et al., 2002, 2005; Kim et al., 2011). In fact, PFC dysfunction prior to substance use is thought to increase vulnerability to developing a substance use disorder (Goldstein and Volkow, 2011) and may promote relapse in individuals with prior SUDs.

On a cellular level, repeated cocaine self-administration in rats results in reduced excitability of layer V prefrontal pyramidal cells, which is further exacerbated in a subset of animals showing compulsive drug-seeking (Chen et al., 2013). Such reductions in deep layer pyramidal cell excitability following cocaine exposure are characterized by a reduced ability to enter “up-states,” whereby action potentials are promoted by synchronous excitatory inputs (Trantham et al., 2002). To date, no studies have examined the effects of the abused cocaine-like synthetic cathinone 3,4-methylenedioxypyrovalerone (MDPV) on prefrontal excitability. The effects of methamphetamine on mPFC function appear to be somewhat complex where self-administration increases the amplitude of excitatory currents elicited in deep pyramidal cells (Pena-Bravo et al., 2019) in rats, decreases α-amino-3-hydroxy-5-methyl-4-isoxazolepropionic acid/*N*-methyl-D-aspartate (AMPA/NMDA) ratios in mice (Mishra et al., 2017), and increases *in vivo* basal firing rates of putative deep layer pyramidal neurons (Parsegian et al., 2011). Further, methamphetamine self-administration induced decreases in glutamate release probability from pre-synaptic terminals targeting D1-expressing layer V pyramidal neurons in the PFC have been observed (González et al., 2019), which would suggest that methamphetamine reduces the downstream excitability of type I cells.

Studies on the effects of opioids on prefrontal neuronal excitability are extremely sparse. Of the few studies conducted on single cell activity, heroin decreases AMPA/NMDA ratios in layer V pyramidal neurons (Van den Oever et al., 2008), mainly through reductions in AMPA receptor activity and expression. Similarly, non-contingent morphine exposure reduces cellular excitability through reductions in mPFC AMPA GluA1 receptor expression and is also reflected in reductions in expression of the activity-related immediate early gene c-fos in this region (Piao et al., 2017). Taken together, these studies highlight that, in general, opioids decrease prefrontal pyramidal cell excitability (Hearing et al., 2012), yet their effects on pyramidal cell subtypes remain unknown.

Binge-like ethanol intake upregulates NMDA-receptor-mediated post-synaptic plasticity in deep pyramidal cells (Kroener et al., 2012; Klenowski et al., 2016), increases dendritic arborization of layer V pyramidal cells, and increases the frequency of spontaneous excitatory post-synaptic potentials (Klenowski et al., 2016). Following chronic intermittent ethanol exposure, AMPA/NMDA ratios of mPFC layer V pyramidal neurons are significantly increased (Kroener et al., 2012), further suggesting enhanced prefrontal excitability of layer V pyramidal neurons. Similarly, ethanol withdrawal has been shown to increase the excitatory transmission of layer 2/3 pyramidal neurons (Varodayan et al., 2018), which are known to communicate directly with layer V pyramidal cells. Taken together, these effects suggest that ethanol has not only direct effects on layer V prefrontal neurons but also indirect effects on inputs targeting these cells.

Given that no studies to date have examined the effects of drugs on layer V pyramidal subtypes, the overarching goal of the study was to determine the effects of various abused substances on prefrontal layer V pyramidal cell excitability and to determine whether these effects were preferential for different cellular subtypes. We hypothesized that, given the role of type II pyramidal cells in controlling compulsive behaviors (Kim et al., 2017), all tested drugs of abuse would decrease activity of type II pyramidal cells over type I cells. In our study, we employed a repeated once daily regimen of administration of either saline, heroin, methamphetamine, ethanol, cocaine, or MDPV (i.p.) for five consecutive days. Following the last exposure, whole-cell physiological recordings were conducted from layer V pyramidal cells within the prefrontal cortex. All pyramidal cells were distinguished as type I or type II based on baseline physiological characteristics.

## Materials and Methods

### Animals

C57BL/6 wildtype mice used in all experiments were bred in-house and housed in a temperature-controlled vivarium (22–24°C) on a reverse light/dark cycle (14 h/10 h light/dark, lights off at 0700). All mice were given standard chow and water *ad libitum*. All experiments conducted were approved by the Institutional Animal Care and Use Committee of Arizona State University. All animals were single housed for a minimum of 2 days prior to the start of i.p. drug injections.

### Intraperitoneal Injections and Slice Preparation

**FIGURE 1:**
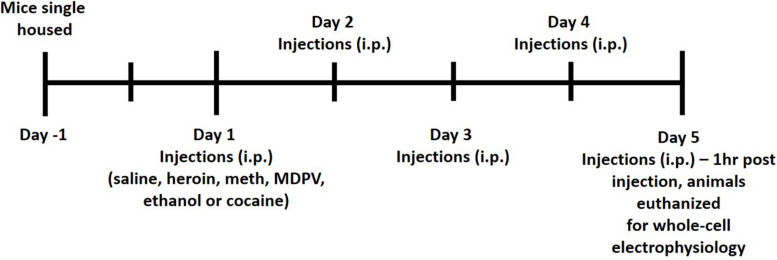
A timeline of experimental procedures.

All animals received i.p. injections of either saline, ethanol (2 g/kg), methamphetamine (meth, 1 mg/kg), cocaine (10 mg/kg), heroin (2 mg/kg), or MDPV (2.5 mg/kg) for five consecutive days. All drugs were prepared for injection volumes of 0.1 mL per 10 g of body mass. Commercial sources for drugs were as follows: ethanol (Koptec), methamphetamine (Sigma-Aldrich), heroin (Cayman Chemical), and MDPV (Laboratory Supply USA). All doses used in the current study were chosen based on previous literature demonstrating drug-induced conditioned place preference (CPP) in mice (Martin et al., 2000; Thanos et al., 2005; Schlussman et al., 2008; Karlsson et al., 2014; Xu et al., 2021). All injections were given between 3 and 4 h following lights off. One hour following the fifth injection (on day 5), animals were euthanized for whole-cell patch clamp electrophysiology. A timeline of this procedure is shown in Figure 1. Animals were anesthetized with carbon dioxide (CO_2_), rapidly decapitated, and brains were removed and immersed in ice-cold carbogen (95% O_2_/5% CO_2_) saturated artificial cerebrospinal fluid (aCSF) cutting buffer containing (in mmol/L): NaCl, 120; NaHCO_3_, 25; dextrose, 10; KCl, 3.3, NaH_2_PO_4_, 1.3; CaCl_2_, 1.8; MgCl_2_, 2.4. Osmolarity was adjusted to 290 ± 5 and pH was adjusted to 7.40 ± 0.01. Brains were then transferred to the cutting chamber of a vibrating tissue slicer (Leica model VT1000S), and coronal slices containing the mPFC were prepared. Slices were cut at a thickness of 300 μm and were taken from approximately 200–1,400 μm caudal to the frontal pole. Slices were then transferred to a holding chamber filled with a recording aCSF continuously saturated with carbogen containing (in mmol/L): NaCl, 120; NaHCO_3_, 25; dextrose, 10; KCl, 3.3, NaH_2_PO_4_, 1.3; CaCl_2_, 2; MgCl_2_, 1. Osmolarity was adjusted to 290 ± 5 and pH was adjusted to 7.40 ± 0.01. Slices were incubated at 34°C for 45 min and then allowed to cool to room temperature prior to being transferred to the recording chamber. Once transferred to the recording chamber, slices were continuously perfused with recording aCSF at a flow-rate of 1–2 mL/min.

### Electrophysiology

All recordings were conducted from layer V pyramidal neurons located within the infralimbic and prelimbic areas of the mPFC, which were visually identified on an Olympus BX51WI microscope (Tokyo, Japan) using infrared DIC microscopy at 600X magnification. A total of 115 cells (*n* = 51 type I cells and *n* = 64 type II cells) were recorded from in the current study, where 17–21 cells per drug treatment group were obtained. Whole-cell recordings were performed using recording pipets (6–9 MΩ tip resistance) made from thin-walled glass capillary tubes filled with an intracellular solution containing (in mmol/L): K-gluconate, 135; NaCl, 12; K-EGTA, 1; HEPES, 10; Mg-ATP, 2; and tris-GTP, 0.38. Osmolarity was adjusted to 285 ± 5 mOsm and pH was adjusted to 7.30 ± 0.01. Axograph software was used to conduct all recordings, where responses were digitized at 10 kHz and saved onto a hard drive using the Digidata interface (Axon Instruments). All recordings were analyzed offline using Axograph. Experimental protocols used in the current study have been outlined in detail in our previous study (Leyrer-Jackson et al., 2020). Briefly, we used a 15-step depolarization protocol in current clamp to assess rheobase (amount of current required to elicit action potentials). Using the spikes elicited within the rheobase protocol, we examined action potential characteristics including rise time, decay time, and amplitude. These data were used to generate input/output curves (i.e., the number of spikes elicited by depolarizing currents), as well as to calculate action potential threshold. Cellular capacitance and membrane potential were also measured to examine whether drug exposure alters baseline cellular properties. Lastly, we explored potential differences in these measurements between type I and type II pyramidal cells, which were distinguished based on the presence of a prominent “sag” in response to a 125 pA hyperpolarizing current, and by initial firing of doublets (found in type I cells only). Both criteria for cell type identification have been used in previous studies (Dembrow et al., 2010; Spindle and Thomas, 2014; Leyrer-Jackson and Thomas, 2018).

### Data Analysis

**FIGURE 2:**
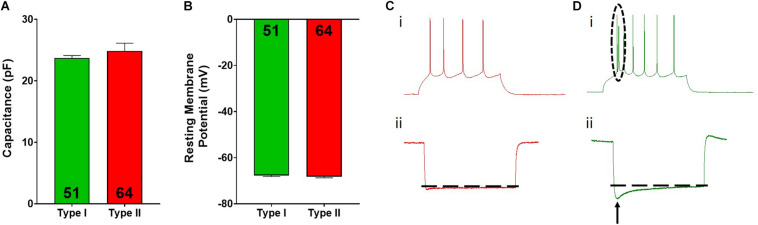
Type I and type II pyramidal cell electrophysiological characteristics. Type I (green) and type II (red) cells do not differ in cellular capacitance **(A)** or resting membrane potential **(B)**. Representative spiking for type II **(Ci)** and type I **(Di)** cells is shown. The dotted circle in **Di** highlights the spiking doublet elicited in type I cells. Representative hyperpolarization traces for type II **(Cii)** and type I **(Dii)** cells are shown, where type I cells display a large “sag” indicated by the arrow. Inset numbers in **(A,B)** represent the number of cells analyzed.

All data analyses were conducted using GraphPad Prism 9.0. Comparisons between groups were made using analyses of variance (ANOVAs), *post hoc* comparisons, *t*-tests, or linear regression models. Briefly, two-way ANOVA Bonferroni *post hoc* comparisons were used to compare all six treatment groups for each variable. T-tests were used to assess differences in type I and type II cell types within treatment group (Figure 4D) or across measurements (Figures 2A,B). Linear regression analyses were used to assess differences in slope between treatment groups across the input/output curves described in Figures 5, 6. Specific analyses used for comparisons are depicted within the results and figure legends, where appropriate. All data are presented as mean ± SEM. *P*-values of less than 0.05 were considered statistically significant.

**FIGURE 3:**
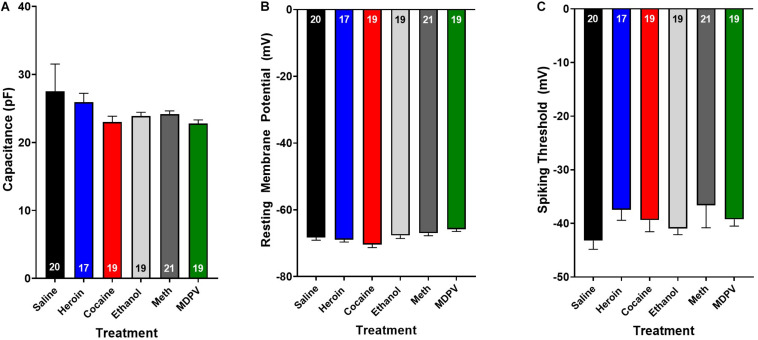
Drug exposure does not alter basic cellular physiology. Exposure to heroin (blue), cocaine (red), ethanol (light gray), meth (dark gray), or MDPV (green) does not alter cellular capacitance **(A)**, resting membrane potential **(B),** or spiking threshold **(C)** of all cells recorded (grouped across type I and type II cells). Inset numbers in histograms represent the number of cells analyzed.

**FIGURE 4:**
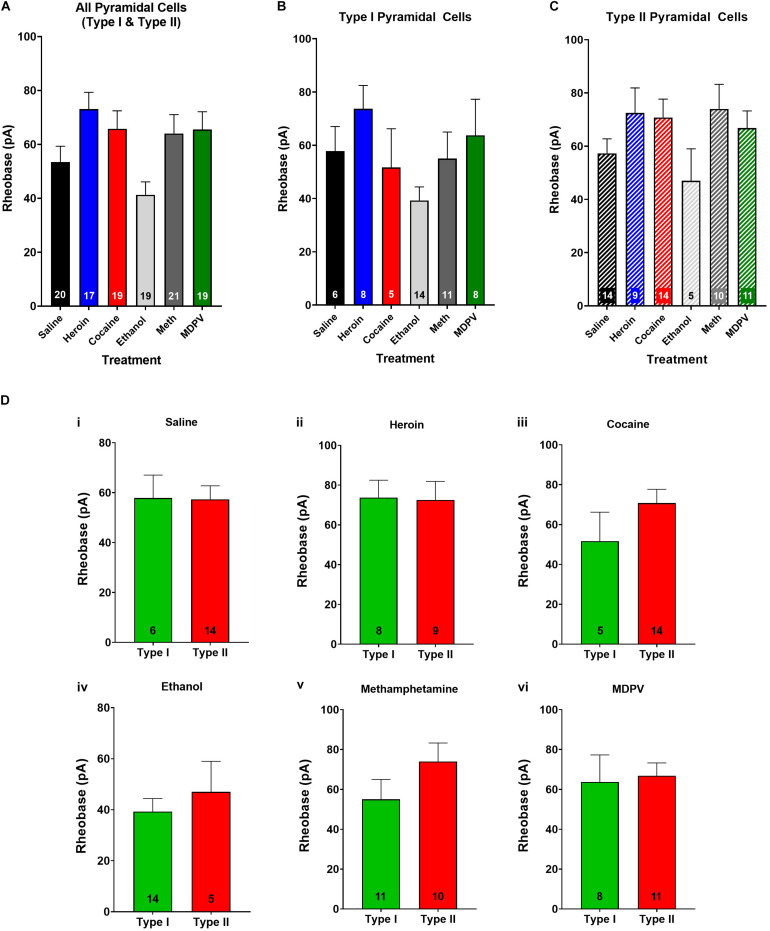
Rheobase of layer V pyramidal cells is not altered by drug treatment and does not differ between pyramidal subtypes. **(A)** Drug exposure does not alter the rheobase of layer V pyramidal cells, regardless of subtype **(B,C)**. The rheobase of type I and type II cells did not differ within saline **(Di)**, heroin **(Dii)**, cocaine **(Diii)**, ethanol **(Div)**, methamphetamine **(Dv)**, or MDPV **(Dvi)** treated animals. Numbers within histogram bars represent the number of recorded cells.

**FIGURE 5:**
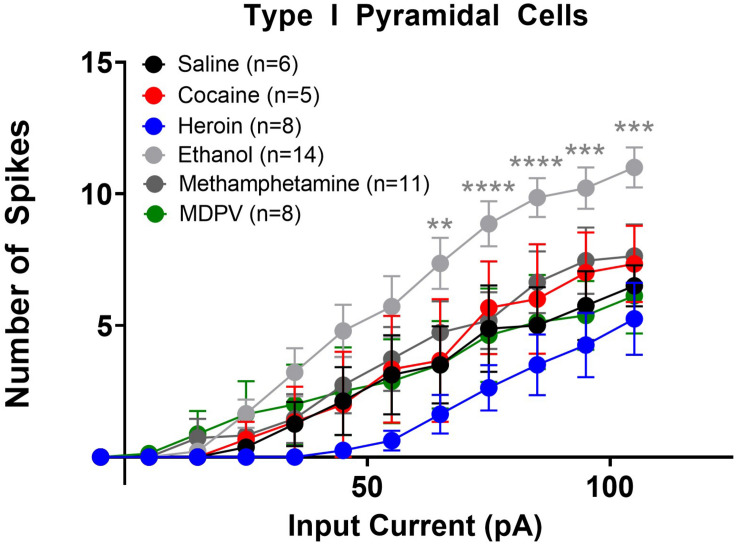
Ethanol increases the excitability of type I layer V pyramidal cells. Ethanol significantly increased the number of action potentials elicited with higher depolarizing currents in type I cells, relative to saline. Specifically, ethanol significantly increases the number of action potentials elicited by currents 65–105 pA. Cocaine, heroin, methamphetamine, and MDPV had no effect on the number of depolarization-induced action potentials in type I cells. **, ***, and **** indicate *p* < 0.01, *p* < 0.001, and *p* < 0.0001 saline versus ethanol, respectively. Cell numbers are indicated within figure legends.

**FIGURE 6:**
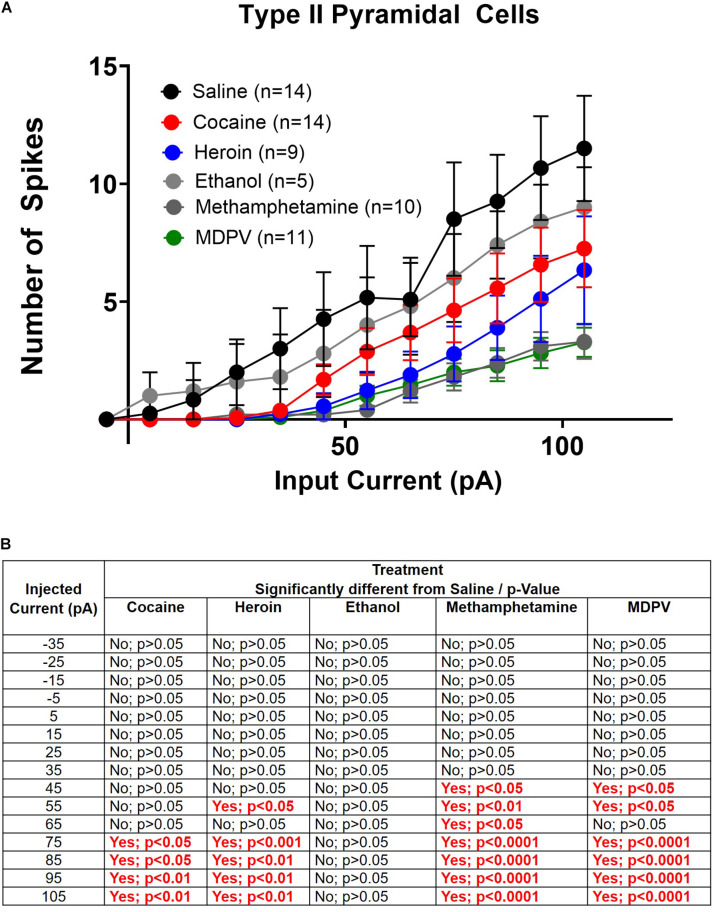
Drug induced changes in type II pyramidal cell excitability. **(A)** Input/output curves of type II cells for all drug groups are shown. **(B)** Significance at each injected current for each drug group relative to saline. Cell numbers are indicated within figure legends in **(A)**.

## Results

Overall, pyramidal cells had an average cellular capacitance of 24.5 ± 0.7 pA, resting membrane potential of −68 ± 0.35 mV, and spiking threshold of −40.3 ± 0.6 mV. Type I and type II layer V pyramidal cells were characterized based on their baseline physiological properties including the presence of a hyperpolarization activated cationic current (“sag”) and/or the presence of a spike afterdepolarization (ADP)/spiking doublet. As shown by others (Dembrow et al., 2010; Spindle and Thomas, 2014; Leyrer-Jackson and Thomas, 2017, 2018, 2019), type I and type II cells did not differ in cellular capacitance (type I: 23.7 ± 0.4 pA, type II: 24.8 ± 1.3 pA; *p* > 0.05; Figure 2A) or resting membrane potential (type I: −67.7 ± 0.5 mV, type II: −68.2 ± 0.5 mV; *p* > 0.05; Figure 2B). Representative traces depicting action potential firing of a type I and type II cell are shown in Figures 2C,D. The spiking doublet exhibited by type I cells is highlighted. Hyperpolarization traces are also shown for both cell types (Figures 2Cii,Dii), where type I cells display a prominent sag (indicated by the arrow in Figure 2Dii). An ANOVA, where treatment was considered a factor revealed that drug treatment had no effect on cellular capacitance, resting membrane potential, or spiking threshold (Figure 3A–C; *p* > 0.05).

### Drug Exposure Does Not Alter Rheobase Relative to Saline

When type I and type II cells were grouped together, an ANOVA revealed a significant effect of treatment on rheobase (*F*_4_,_90_ = 3.8; *p* < 0.01; Figure 4A). However, a *post hoc* comparison revealed that drug exposure had no significant effects on rheobase relative to pyramidal cells of saline-treated animals (Figure 4A). Furthermore, when type I and type II cells were analyzed separately, an ANOVA showed no significant effects of treatment on rheobase in either cell type (*p* > 0.05; Figures 4B,C). No differences in rheobase of type I and type II cells within treatment groups were observed (*p* > 0.05; Figure 4D).

### Drug Exposure Alters Depolarization-Induced Action Potentials of Type I and Type II Cells

We next assessed changes in action potential output induced by stepped depolarizing currents. Specifically, we assessed the number of action potentials elicited by varying amplitudes of injected currents. When all cells (type I and type II) were grouped together, a two-way ANOVA with treatment and input current amplitude as factors revealed significant effects of current input (*F*_14_,_1635_ = 78.8; *p* < 0.0001), treatment (*F*_5_,_1635_ = 30.8; *p* < 0.0001), as well as a significant interaction between these two factors (*F*_70_,_1635_ = 1.9; *p* < 0.0001). *Post hoc* comparisons of the number of action potentials elicited at each current revealed that larger depolarizing currents (i.e., at or above 75 pA) elicited significantly fewer action potentials in pyramidal cells of animals treated with heroin, meth, and MDPV relative to saline (*p* < 0.05). When separated by cell type, a comparison of type I cells across drug treatment groups showed that heroin, meth, cocaine, and MDPV had no significant effects on the number of action potentials elicited at any current (*p* > 0.05; Figure 5). However, ethanol significantly increased the number of action potentials elicited in type I cells relative to saline, with depolarizing currents of 65 pA (*t*_690_ = 3.2; *p* < 0.01), 75 pA (*t*_690_ = 3.3; *p* < 0.01), 85 pA (*t*_690_ = 4.0; *p* < 0.001), 95 pA (*t*_690_ = 3.7; *p* < 0.01), and 105 pA (*t*_690_ = 3.7; *p* < 0.01). These results are shown in Figure 5.

Next, we explored drug-induced changes in depolarization-induced action potential number within type II cells. A two-way ANOVA, where treatment and input current amplitude were considered factors, revealed significant effects of current input (*F*_14_,_885_ = 28.1; *p* < 0.0001), treatment (*F*_5_,_855_ = 22.7; *p* < 0.0001), and a significant interaction between these two factors (*F*_70_,_855_ = 1.4; *p* < 0.05) (Figure 6A). *Post hoc* comparisons of the number of action potentials elicited at each current revealed that greater amplitude depolarizing currents elicited significantly fewer action potentials in type II pyramidal cells of animals treated with heroin, cocaine, meth, and MDPV relative to saline (*p* < 0.05). These results are highlighted within the table in Figure 6B. However, ethanol did not alter the number of action potentials elicited by any depolarizing current compared to saline within type II cells (*p* > 0.05; Figure 6B).

Linear regression analyses assessing the slope of input/output curves were conducted for type I and type II cells from animals treated with all drugs using the same cell types from saline-treated animals as a comparison group. For type I cells, we found that heroin treatment significantly reduced the slope of the fitted linear regression (*F*_1_,_118_ = 70.4; *p* < 0.05). However, ethanol significantly increased the slope of the fitted linear regression (*F*_1_,_208_ = 452.9; *p* < 0.0001). We also found that heroin, cocaine, methamphetamine, and MDPV all significantly reduced the slope of the fitted linear regression in type II cells (heroin: *F*_1_,_133_ = 49.3, *p* < 0.0001; cocaine: *F*_1_,_238_ = 103.2, *p* < 0.001; methamphetamine: *F*_1_,_148_ = 94.5, *p* < 0.0001; MDPV: *F*_1_,_163_ = 102.2, *p* < 0.0001). These results are depicted within Table 1.

**TABLE 1:** Linear regression analyses results.

Cell type	Drug	F-value	DFn, DFd	Slope	Slope different from zero; *p*-value	Slope different from saline; *p*-value
**Type I**	Saline	80.9	1,118	0.05	Yes; *p* < 0.0001	
	Heroin	70.4	1,118	0.03	Yes; *p* < 0.0001	Yes; *p* < 0.05*
	Cocaine	61.0	1,43	0.06	Yes; *p* < 0.0001	No; *p* > 0.05
	Ethanol	452.9	1,208	0.09	Yes; *p* < 0.0001	Yes; *p* < 0.0001****
	Methamphetamine	138.8	1,163	0.02	Yes; *p* < 0.0001	No; *p* > 0.05
	MDPV	39.5	1,88	0.06	Yes; *p* < 0.0001	No; *p* > 0.05
**Type II**	Saline	94.3	1,178	0.09	Yes; *p* < 0.0001	
	Heroin	49.3	1,133	0.04	Yes; *p* < 0.0001	Yes; *p* < 0.0001****
	Cocaine	103.2	1,238	0.06	Yes; *p* < 0.0001	Yes; *p* < 0.001***
	Ethanol	71.6	1,73	0.07	Yes; *p* < 0.0001	No; *p* > 0.05
	Methamphetamine	94.51	1,148	0.02	Yes; *p* < 0.0001	Yes; *p* < 0.0001****
	MDPV	102.2	1,163	0.02	Yes; *p* < 0.0001	Yes; *p* < 0.0001****

### Drug Exposure Alters Action Potential Amplitude but Not Rise Time or Decay Time of Layer V Pyramidal Cells

**FIGURE 7:**
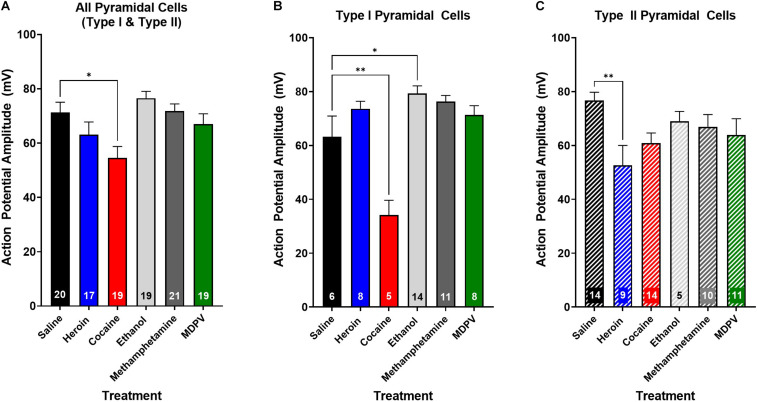
Drug exposure differentially alters action potential amplitude. **(A)** Cocaine exposure significantly reduces action potential amplitude of layer V pyramidal cells compared to saline. **(B)** Cocaine significantly reduces the action potential amplitude of type I cells relative to saline, whereas ethanol significantly increases action potential amplitude. **(C)** Heroin significantly reduces action potential amplitude of type II, but not type I cells, relative to saline. Numbers within histogram bars represent the number of recorded cells. * Indicates *p* < 0.05 vs. saline; ** indicates *p* < 0.01 vs. saline.

We next assessed whether drug exposure had any effects on action potential rise time or decay time. An ANOVA, where drug treatment was considered a factor, revealed no effects on action potential rise time (*p* > 0.05) or decay time (*p* > 0.05) when all pyramidal cells were grouped across cell types. Additionally, drug treatment had no effect on the rise time or decay time of type I (*p* > 0.05) or type II cells (*p* > 0.05), when grouped individually. Interestingly, drug exposure had a significant effect on action potential amplitude (*F*_5_,_106_ = 1.0; *p* < 0.01; Figure 7A), and *post hoc* analyses revealed that the action potential amplitude of pyramidal cells from animals treated with cocaine was significantly less than that of animals treated with saline (*t*_106_ = 4.6; *p* < 0.05; Figure 7A). Furthermore, this effect appeared to be driven primarily by type I cells (Figure 7B), as a significant effect of drug treatment on action potential amplitude in type I cells (*F*_4_,_40_ = 11.7; *p* < 0.0001; Figure 7B) was observed, and *post hoc* comparisons revealed that type I cells of animals treated with cocaine had a significantly lower action potential amplitude than type I cells of animals treated with saline (*t*_40_ = 5.4; *p* < 0.01; Figure 7B). Further, type I cells of animals treated with ethanol had a significantly higher action potential amplitude than type I cells of animals treated with saline (*t*_40_ = 4.1; *p* < 0.05; Figure 7B). An ANOVA comparing the action potential amplitude of type II cells between treatment groups revealed a significant effect of drug treatment (*F*_4_,_43_ = 4.0; *p* < 0.01; Figure 7C), and *post hoc* analyses revealed that type II cells of animals treated with heroin had a significantly lower action potential amplitude than type II cells of animals treated with saline (*t*_43_ = 5.3; *p* < 0.01; Figure 7C), an effect that was not observed in type I cells (*p* > 0.05; Figure 7B).

## Discussion

In the present study, we explored the effects of repeated administration of heroin, cocaine, MDPV, ethanol, and methamphetamine on the physiological characteristics and excitability of layer V pyramidal cells within the mPFC. Here we report that drug exposure changes the excitability of layer V pyramidal cells by altering the number of spikes evoked with increasing current injections, whereby each drug class has preferred effects on either type I or type II cells. Specifically, ethanol increases the excitability of type I pyramidal cells as measured by the number of depolarization induced spikes relative to type I cells of saline-treated animals, whereas cocaine, heroin, methamphetamine, and MDPV decrease the number of depolarization-induced spikes in type II cells, relative to type II cells in animals treated with saline. Furthermore, drug treatment significantly alters the amplitude of action potentials in a subtype-specific manner. We show that cocaine significantly decreases, while ethanol significantly increases, action potential amplitude in type I, but not type II cells. Further, heroin significantly reduces spike amplitude in type II cells. Together, these results suggest that heroin, cocaine, MDPV, and methamphetamine reduce commissural connectivity of the mPFC by reducing type II excitability, while ethanol increases the excitability of type I cells targeting subcortical structures.

### Ethanol Enhances mPFC Type I Pyramidal Cell Excitability

The effects of ethanol on pyramidal excitability have been studied in a variety of pyramidal cells within the prefrontal cortex including layer II/III pyramidal cells (Pleil et al., 2015) as well as deep layer V/VI pyramidal cells (Kroener et al., 2012; Klenowski et al., 2016). Long-term ethanol exposure and chronic intermittent ethanol exposure have been shown to increase synaptic plasticity through enhanced spontaneous excitatory post-synaptic current frequency (Klenowski et al., 2016) and increased AMPA/NMDA ratios (Kroener et al., 2012). These results have been further solidified with correlative changes in enhanced dendritic arborization (Pleil et al., 2015; Klenowski et al., 2016) and increased numbers of mushroom-type spines generally associated with long-term potentiation (Kroener et al., 2012). Following ethanol exposure, recordings from layer II/III pyramidal cells within the mPFC have also revealed enhanced excitatory post-synaptic potential amplitudes and reductions in spontaneous inhibitory post-synaptic currents (Pleil et al., 2015), suggesting enhanced pyramidal cell excitability as a result of reductions in inhibitory control. Aside from its effects on glutamatergic pyramidal cells, ethanol exposure results in reduced activity of fast-spiking interneurons within the prefrontal cortex (Hughes et al., 2020; Li et al., 2021). Specifically, both parvalbumin (PV+) and somatostatin (SOM+) expressing fast-spiking interneurons show reduced excitability following ethanol exposure (Hughes et al., 2020; Li et al., 2021), thus rendering pyramidal cells more excitable due to reduced inhibitory tone. These reductions have been reported for neurons within layer V, where their inhibitory control of layer V pyramidal cells aides in glutamatergic regulation of proper network activity (Hughes et al., 2020; Li et al., 2021). Interestingly, PV+ and SOM+ fast-spiking interneurons preferentially target type I pyramidal cells where their inhibitory control supersedes that exerted on type II pyramidal cells (Anastasiades et al., 2018). In the present study, we report that ethanol exposure increases the excitability of type I but not type II cells. Such an effect may be caused by reduced fast-spiking interneuron activity induced by ethanol exposure. However, additional studies exploring both pre- and post-synaptic effects of ethanol on pyramidal neurons are warranted. Together with previous studies, our results suggest that enhancement in layer V excitability by ethanol is likely driven by type I pyramidal cells.

### Drug Induced Reductions in mPFC Type II Pyramidal Cell Excitability

We report here that cocaine, heroin, MDPV, and methamphetamine reduce the excitability of type II pyramidal cells, where higher current inputs yielded less action potentials than that observed in saline-treated animals. While studies examining the effects of these drug classes on mPFC pyramidal cell excitability are relatively sparse, some have shown that morphine decreases the excitability of pyramidal cells in response to excitatory inputs from the mediodorsal thalamus as well as locally applied glutamate (Giacchino and Henriksen, 1998). Interestingly, in this study, morphine had less of an effect on pyramidal cell integration of basolateral amygdala and hippocampus, suggesting that opioids likely alter layer V pyramidal cell integration of specific inputs, such as from the thalamus. Moreover, type II pyramidal cells are more responsive to thalamic inputs compared to type I (Collins et al., 2018) and thus, we hypothesize that opioids likely reduce type II excitability in part by reducing thalamic drive preferentially targeting this layer V subpopulation.

Other drug classes, such as cocaine and MDPV, act at the presynaptic dopamine transporter to inhibit dopamine reuptake. Until now, the effects of MPDV on whole-cell neuronal excitability including rheobase and input/output curves had not been explored. However, a preclinical neuroimaging study demonstrated that acute administration of MDPV in rats significantly reduced functional connectivity between the mPFC and other cortical regions, as well as with the dorsal and ventral striatum, in a dopamine receptor-independent manner (Colon-Perez et al., 2016). In the context of the present study, these observations suggest that MDPV-induced reductions of intracortical mPFC functional connectivity may be a direct result of reduced mPFC type II pyramidal cell excitability, whereas reductions in corticostriatal functional connectivity are likely a result of MDPV actions outside the mPFC. Others have shown that cocaine exposure reduces excitability of layer V pyramidal neurons within the mPFC (Dong et al., 2005; Chen et al., 2013). Specifically, cocaine self-administration by rats results in a significant reduction in the number of action potentials elicited by a depolarizing current (Chen et al., 2013), and rescuing action potential output using optogenetics prevents cocaine seeking. However, others have shown that cocaine exposure reduces voltage-gated potassium currents within mPFC pyramidal cells, resulting in increased membrane excitability (Dong et al., 2005). Aside from pyramidal neurons, five consecutive days of i.p. cocaine exposure (similar to the procedures used in the current study) reduces the number of action potentials elicited by large current inputs in fast spiking interneurons (Slaker et al., 2018), an effect that would likely increase pyramidal cell excitability. It is of note that these reductions in GABAergic neuron activity were identified 3 days following the cessation of cocaine injections (Dong et al., 2005), where cellular effects are known to be vastly different than during cocaine exposure (Van den Oever et al., 2010). Cocaine has also been shown to reduce inhibitory signaling mediated by various G-protein coupled receptors as well as via indirect activation of D_2_ dopaminergic receptors. Dopaminergic receptor expression is known to differ between layer V pyramidal subtypes where type I cells express D2 receptors and type II do not (Gee et al., 2012). Thus, cocaine-induced effects on D2 receptors would be concentrated at type I cells, likely promoting type I neuronal excitation. However, we did not observe cocaine-induced excitability increases in type I pyramidal neurons, suggesting that other compensatory mechanisms may play a role in this process. Given that none of the studies described above explored differences in effects of cocaine on type I vs. type II cells, it is hard to decipher whether these effects were driven by one of the pyramidal cell subtypes, or whether these effects were generalized across layer V pyramidal cells. Here we observed a significant reduction in the number of action potentials elicited by large current injections in type II cells, an effect that was not observed when both layer V pyramidal subtypes were grouped together, and therefore it is likely that the studies denoted above would have markedly different results if pyramidal subtypes were explored. Overall, reductions in type II cell excitability could hinder the ability of the commissural connectivity to maintain reverberant activity, preventing information from being held during executive functioning tasks, and thus, result in compulsivity, loss of behavioral control, and drug relapse (Van den Oever et al., 2010; Kim et al., 2017).

Lastly, we found that the action potentials elicited in layer V pyramidal cells of animals treated with cocaine had significantly lower amplitudes than those of animals treated with saline, an effect that appeared to be driven primarily by type I cells. Prior studies have demonstrated that cocaine withdrawal (i.e., 3 days following the last i.p. cocaine exposure) leads to enhanced sodium channel phosphorylation (Hu et al., 2005), which can lead to a reduction in activation of these channels (Zhang et al., 1998). In medium spiny neurons (MSNs) of the nucleus accumbens core, cocaine withdrawal induces reductions in action potential amplitude, which are due to reductions in sodium channel activation (Zhang et al., 1998). Thus, while this mechanism was previously identified in MSNs, it is possible that reductions in sodium channel activation by cocaine exposure may lead to reductions in action potential amplitude as reported in the current study. Further, it is feasible that type I pyramidal cells are more prone to sodium channel modifications that would give rise to the effects observed on type I cells, relative to type II cells. However, further studies exploring this mechanism and its effects on downstream targets are warranted to fully understand how these alterations could contribute to enhanced drug seeking behaviors and prefrontal dysfunction.

### Limitations of the Current Study

In the current study, we report the effects of cocaine, heroin, methamphetamine, ethanol, and MDPV on mPFC pyramidal cell excitability. However, within the study, we utilized only one dose per drug, which were chosen based on the known ability of that dose to elicit CPP (Martin et al., 2000; Schlussman et al., 2008; Karlsson et al., 2014; Pandy et al., 2018). Thus, because varying dosages are known to elicit different strengths of drug-induced CPP, where low dosages fail to elicit drug-induced CPP and high doses can be aversive, there are likely dose-dependent effects on pyramidal cell excitability that we did not explore in the current study. Thus, we cannot exclude the idea that lower and higher doses than those used in the current study could elicit different effects on pyramidal cell excitability than observed here. Future studies exploring dose-dependent effects of these substances on pyramidal cell excitability would greatly enhance our understanding of how these substances alter prefrontal physiology that may give rise to differences in disruption of mPFC function.

### Implications for Medial Prefrontal Function

Given the differential effects of ethanol and other drugs of abuse on type I and type II pyramidal cells, respectively, it is conceivable that each drug alters prefrontal neuronal function in distinct ways to impair executive functioning. Within the PFC, layer V pyramidal neurons are critical regulators of synaptic information, where they receive “feed-back” of contextual information within the apical tufts of layer I neurons, and “feed-forward” environmental information is delivered to synapses on basal dendrites of layer V neurons (Larkum et al., 2009). When both feed-back and feed-forward information is received synchronously by layer V pyramidal cells, they act as coincidence detectors which can lead to local synaptic plasticity as well as changes in downstream target neurons (Larkum et al., 2009). Given the differences in morphology, downstream connectivity, and preferred excitability frequencies of cortical pyramidal neurons, it is likely that layer V pyramidal cell subtypes contribute to distinct aspects of executive functioning, although this phenomenon is far from understood. Results of the current study suggest that ethanol promotes subcortical excitation through enhanced excitability of type I cells, which may result in inappropriate synaptic plasticity in subcortical structures such as the striatum, that could promote ethanol seeking. Additionally, we demonstrate that heroin, cocaine, MDPV, and methamphetamine reduce type II pyramidal cell excitability. With known roles of these cell types as temporal integrators (Larkum et al., 2009), we hypothesize that reduced excitability of type II cells may hinder top-down and bottom-up processing, ultimately impairing the ability to appropriately integrate previously known information (top-down) as well as ongoing environmental information (bottom-up). Such deficits may ultimately lead to impaired executive functioning, promoting compulsivity and continued drug seeking. Although the precise function and differences between layer V pyramidal subtypes are far from understood, the findings outlined in the current study provide evidence that different drugs of abuse differentially affect layer V pyramidal subtypes, which may ultimately give rise to compulsivity and inappropriate synaptic plasticity that may underlay SUDs. Future studies exploring additional mechanisms for how different drugs of abuse alter layer V pyramidal subtypes and prefrontal connectivity may prove beneficial for understanding drug-seeking behaviors and relapse propensities.

## Data Availability Statement

The original contributions presented in the study are included in the article/supplementary material. Further inquiries can be directed to the corresponding author.

## Ethics Statement

The animal study was reviewed and approved by the Arizona State University Animal Care and Use Committee.

## Author Contributions

JL-J designed and conducted studies in the manuscript, conducted data analyses, and drafted the original manuscript. LH participated in data collection and data interpretation and contributed with editing the manuscript. MFO contributed to study design, manuscript preparation, and data interpretation. All authors contributed to the article and approved the submitted version.

## Conflict of Interest

The authors declare that the research was conducted in the absence of any commercial or financial relationships that could be construed as a potential conflict of interest.

## Publisher’s Note

All claims expressed in this article are solely those of the authors and do not necessarily represent those of their affiliated organizations, or those of the publisher, the editors and the reviewers. Any product that may be evaluated in this article, or claim that may be made by its manufacturer, is not guaranteed or endorsed by the publisher.
